# Dynamic re-immunization of off-treatment childhood cancer survivors: An implementation feasibility study

**DOI:** 10.1371/journal.pone.0191804

**Published:** 2018-02-01

**Authors:** Jennifer H. Han, Kathryn M. Harmoney, Elif Dokmeci, Jacqueline Torrez, Cathy M. Chavez, Loretta Cordova de Ortega, John F. Kuttesch, Martha Muller, Stuart S. Winter

**Affiliations:** 1 Department of Pediatrics, The University of New Mexico Health Sciences Center, 1 University of New Mexico, Albuquerque, New Mexico, United States of America; 2 Department of Pediatrics, Division of Hematology/Oncology, University of Iowa Stead Family Children’s Hospital, 200 Hawkins Drive, Iowa City, Iowa, United States of America; 3 Department of Pediatrics, Division of Allergy/Immunology, The University of New Mexico Health Sciences Center, 1 University of New Mexico, Albuquerque, New Mexico, United States of America; 4 Department of Pediatrics, Division of Hematology/Oncology, The University of New Mexico Health Sciences Center, 1 University of New Mexico, Albuquerque, New Mexico, United States of America; 5 Department of Pediatrics, Division of Infectious Diseases, The University of New Mexico Health Sciences Center, 1 University of New Mexico, Albuquerque, New Mexico, United States of America; 6 Cancer and Blood Disorders Program, Children’s Hospitals and Clinics of Minnesota, 2525 Chicago Avenue South, Minneapolis, Minnesota, United States of America; Pennsylvania State University, UNITED STATES

## Abstract

There are no universally approved re-vaccination guidelines for non-transplant pediatric cancer survivors. We hypothesized that by utilizing a response-based re-vaccination schedule, we could tailor vaccine schedules in off-treatment cancer survivors. Pre-vaccination antibody levels were obtained in 7 patients at an average of 20 days after the end of treatment date. In those without protective antibody levels, we administered vaccines 3 months after completion of treatment. Revaccinating patients 3 months after the end of treatment date resulted in protective antibody levels for most vaccines. We showed, on a preliminary basis, that vaccinating non-transplanted pediatric cancer survivors can be dynamically implemented in children with recovering immune function.

## Introduction

Vaccination against infectious diseases plays an integral role in pediatric medical care, and when given on a well-defined schedule, immunization efficacy is almost assured in children who have normal immune function. In contrast, children treated with chemotherapy for childhood malignancies often develop acquired immunological defects in both cell-mediated and humoral immunity, which results in decreased measurable vaccine protection [1–3]. Although re-immunization consensus criteria exist for children who have undergone bone marrow transplantation (BMT) [3], there are no universally approved revaccination guidelines for non-transplanted childhood cancer survivors [2, 4, 5]. For the vast majority of children who receive cytotoxic therapies, but do not require BMT, the lack of re-immunization guidelines creates confusion among healthcare providers regarding best practices for vaccine protection [5].

Quantitative immunologic recovery in this population has been shown to generally occur within six months to one year after completion of chemotherapy [1, 2, 6, 7]. However, there are no consensus guidelines on when to re-vaccinate. Recently, the Infectious Disease Association of America recommended re-immunization at 3 months following cessation of chemotherapy [8]. In contrast, Ruggiero and colleagues recommended delay of live vaccines until 6 months from the end of treatment (EOT) date [9]. Several single institutional studies have evaluated response to vaccinations at varying times in pediatric cancer patients in remission, including up to 12 months after completion of chemotherapy, with generally favorable results [2, 4, 5, 10]. To address these gaps in knowledge, we hypothesized that by utilizing a prospective, response-based revaccination schedule, we could safely implement personalized immunization schedules in post-therapy, non-transplanted childhood cancer survivors. Our findings suggest that immune function in off-therapy patients is more robust than previously thought.

## Materials and method

The study was conducted between March 2014 and August 2016. Participants were enrolled from the pediatric hematology/oncology clinic at the University of New Mexico (UNM) Health Sciences Center in Albuquerque, NM. Eligibility criteria included completion of treatment per the Children’s Oncology Group protocols for any child who received at least 6 months of dose-intensified, cytotoxic therapies that were implemented as risk-adjusted, disease-based therapies. Exclusion criteria included BMT, solid organ transplantation, and subjects younger than 2 months of age or greater than 18 years of age. In accordance with the Declaration of Helsinki and the University of New Mexico’s Human Research Review Committee and Human Research Protections Office, the legal guardians for the research participants provided written, informed consent prior to participation in the study. The University of New Mexico’s Human Research Review Committee and Human Research Protections Office specifically approved of this study (Study ID: 13–553).

Pre-vaccination serum antibody levels were obtained via blood draws at an average of 20 days (range of 7–44 days) after the end of EOT date. In patients for whom pre-vaccination antibody (IgG) levels were not protective, we administered FDA-approved vaccines for *Haemophilus influenzae* type b (Hib), diphtheria, tetanus, poliomyelitis, pneumococcus, measles, mumps, and rubella (MMR) 3 months after EOT. Follow-up IgG levels were then obtained at 5–10 weeks following vaccination to assess immune responses. Using standardized measurement criteria, results were analyzed using Clinical Laboratory Improvement Amendments approved techniques (Table 1).

**Table 1:** Data interpretation for protective threshold antibody levels.

Vaccine	Units	Sub-therapeutic	Therapeutic
Hib^1^	ug/mL	< 1.0	≥ 1.0
Tetanus	ug/mL	< 0.1	≥ 0.1
Diptheria	ug/mL	< 0.1	≥ 0.1
Poliovirus	Neutralization titer concentrations	<1:10	≥ 1:10
Pneumococcus	ug/mL	<1.3 in over 70% of serotypes	≥ 1.3 in at least 70% of serotypes
Measles	None	Negative/equivocal response	Positive response
Mumps	None	Negative/equivocal response	Positive response
Rubella	IU/m	<10	>10

^1^ *Haemophilus influenzae* type b

## Results

A total of 7 patients [4 males, 3 females; mean age 7 years (range 6 to 10 years)] were enrolled (Table 2). Six patients had hematologic malignancies, 5 patients with B-cell acute lymphoblastic leukemia (B-ALL) and 1 patient with T-cell acute lymphoblastic leukemia (T-ALL); one patient had high-risk Wilms tumor. All patients had finished the pneumococcal vaccination series prior to diagnosis. Six patients had completed the Hib vaccination prior to diagnosis. Five patients had completed vaccinations for diphtheria, tetanus, poliovirus, and MMR prior to diagnosis (Table 3).

**Table 2:** Patient characteristics.

Patient	Therapy	Diagnosis Age (years)	Enrollment Age (years)	Time from EOT^1^ to post-therapy IgG^2^ levels	Time from EOT to vaccination	Time from vaccination to obtaining IgG levels
1	Diagnosis: B-ALL^3^ Rx^4^: AALL0932 Duration: 38 months Chemotherapy	4.4	7.8	44 days	4 months	9 weeks
2	Diagnosis: T-ALL^5^ Rx: AALL0434 Duration: 38 months Chemotherapy	5.5	8.10	19 days	4 months	8 weeks
3	Diagnosis: B-ALL Rx: AALL1131 Duration: 26 months Chemotherapy	6.5	8.9	12 days	3 months	5 weeks
4	Diagnosis: B-ALL Rx: AALL0932 Duration: 26 months Chemotherapy	8	10.3	30 days	4 months	8 weeks
5	Diagnosis: Wilms Rx: AREN0532 Duration: 7 months Chemo/Radiation	9.1	9.9	35 days	4 months	9 weeks
6	Diagnosis: B-ALL Rx: AALl1131 Duration: 26 months Chemotherapy	7.3	9.8	7 days	3 months	9 weeks
7	Diagnosis: B-ALL Rx: AALL0932 Duration: 38 months Chemotherapy	2.9	6.1	38 days	4 months	10 weeks

1 End of treatment

2 Immunoglobulin G

3 B-cell acute lymphoblastic leukemia

4 COG protocol type

5 T-cell acute lymphoblastic leukemia

**Table 3:** Results of pre-diagnosis vaccination status, post-treatment IgG levels, and post-vaccine IgG levels.

Patient	Pre-diagnosis vaccination status	Infectious Disease	Post-treatment IgG levels	Immune Status	Vaccines given	Post-vaccine IgG levels	Outcome
1	Incomplete	*Diptheria*	0	non-immune		0	non-immune
	Incomplete	*Tetanus*	0	non-immune		0.3	immune
	Incomplete	*Poliovirus*	<1:10	non-immune		<1:10	non-immune
	Incomplete	*Measles*	Negative	non-immune	MMR	Positive	immune
	Incomplete	*Mumps*	Negative	non-immune		Positive	immune
	Incomplete	*Rubella*	9.2	equivocal		>500	immune
	Incomplete	*HiB*	0.3	non-immune		1.1	immune
	Complete	*Pneumo*	29% (4 of 14)	non-immune	PPSV23	86% (12 of 14)	Immune
2	Complete	*Diptheria*	0.1	immune		Not obtained	immune
	Complete	*Tetanus*	0.6	immune			immune
	Complete	*Poliovirus*	>1:10	immune			immune
	Complete	*Measles*	Negative	non-immune	MMR	Positive	immune
	Complete	*Mumps*	Negative	non-immune		Positive	immune
	Complete	*Rubella*	34.5	immune		142.8	immune
	Complete	*HiB*	1.2	immune		Not obtained	immune
	Complete	*Pneumo*	79% (11 of 14)	immune		Not obtained	immune
3	Complete	*Diptheria*	0.1	immune		Not obtained	immune
	Complete	*Tetanus*	0.3	immune			immune
	Complete	*Poliovirus*	>1:10	immune			immune
	Complete	*Measles*	Positive	immune			immune
	Complete	*Mumps*	Positive	immune			immune
	Complete	*Rubella*	>500	immune			immune
	Complete	*HiB*	0.4	non-immune			immune
	Complete	*Pneumo*	21% (3 of 14)	non-immune	PPSV23	50% (7 of 14)	non-immune
4	Complete	*Diptheria*	0	non-immune	Tdap	0.9	immune
	Complete	*Tetanus*	0.1	immune		2.7	immune
	Complete	*Poliovirus*	<1:10	non-immune		Not obtained	inapplicable
	Complete	*Measles*	Negative	non-immune	MMR	Positive	immune
	Complete	*Mumps*	Equivocal	equivocal		Positive	immune
	Complete	*Rubella*	3.1	non-immune		>500	immune
	Complete	*HiB*	0.4	non-immune		Not obtained	inapplicable
	Complete	*Pneumo*	0% (0 of 14)	non-immune	PPSV23	50% (7 of 14)	non-immune
5	Complete	*Diptheria*	0.5	immune		Not obtained	immune
	Complete	*Tetanus*	1.3	immune			immune
	Complete	*Poliovirus*	>1:10	immune			immune
	Complete	*Measles*	Positive	immune			immune
	Complete	*Mumps*	Positive	immune			immune
	Complete	*Rubella*	262.6	immune			immune
	Complete	*HiB*	2.8	immune			immune
	Complete	*Pneumo*	43% (6 of 14)	non-immune	PPSV23	93% (13 of 14)	immune
6	Complete	*Diptheria*	0	non-immune	DTaP	3.4	immune
	Complete	*Tetanus*	0.1	immune		1.2	immune
	Complete	*Poliovirus*	>1:10	immune		>1:10	immune
	Complete	*Measles*	Negative	non-immune	MMR	Negative	non-immune
	Complete	*Mumps*	Negative	non-immune		Negative	non-immune
	Complete	*Rubella*	21.6	immune		338	immune
	Complete	*HiB*	0.6	non-immune		0.4	non-immune
	Complete	*Pneumo*	36% (5 of 14)	non-immune	PPSV23	79% (11 of 14)	Immune
7	Incomplete	*Diptheria*	0.1	immune	DTaP	1.9	immune
	Incomplete	*Tetanus*	0.8	immune		2.7	immune
	Incomplete	*Poliovirus*	>1:10	immune	IPV	>1:10	immune
	Incomplete	*Measles*	Positive	immune	MMR	Equivocal	equivocal
	Incomplete	*Mumps*	Equivocal	equivocal		Negative	non-immune
	Incomplete	*Rubella*	20.1	immune		174.8	immune
	Complete	*HiB*	6.4	immune		6.6	immune
	Complete	*Pneumo*	86% (12 of 14)	immune		93% (13 of 14)	Immune

### Post-chemotherapy antibody levels

In the immediate EOT period, six out of seven (86%) patients had protective anti-tetanus IgG levels (Table 3). Five out of seven (71%) patients had protective anti-rubella and anti-poliovirus IgG levels. Four out of seven (57%) had protective anti-diphtheria and anti-Hib IgG levels. Three out of seven (42%) patients had protective anti-measles antibodies. Two out of seven (29%) patients had protective anti-mumps and anti-pneumococcal antibodies. Patient #1 re-gained protective IgG concentrations against tetanus and Hib without re-vaccination.

### Antibody levels following vaccination

No patient had an adverse effect related to his or her personalized re-vaccination schedule. All patients who received vaccination to diphtheria, tetanus, rubella, and poliovirus achieved protective antibody levels (Table 3). Three out of five (60%) patients who received vaccination to mumps, measles, and pneumococcus achieved an adequate response.

## Discussion

Most children have normally functioning immune systems and develop protective titers against vaccines antigens antecedent to a cancer diagnosis [7, 11, 12]. Treatment with standard chemotherapy significantly interferes with immune function, as demonstrated by diminished humoral and cellular immunity [10, 13, 14]. While there is a more clearly defined process regarding the reconstitution of the immune system in allogenic BMT recipients who receive high-dose chemotherapy [15], much less is known about the extent and duration of immune dysfunction in pediatric patients with childhood cancers who are treated with risk-adjusted chemotherapy [3, 10].

Studies have demonstrated that immunologic recovery in the non-transplant population generally occurs within six months to one year after completion of chemotherapy [1, 2, 6, 7], as demonstrated by patient 1, who re-acquired protective titers against tetanus and Hib without re-vaccination. Further examples of immunologic recovery were also noted in patient 6 against poliovirus and in patient 7 against Hib and pneumococcus. Interestingly, our pilot study demonstrated that at a much earlier median time of three weeks after completion of standard chemotherapy, most children had acceptable antibody levels for several vaccines (Table 3). Our findings indicate that immunologic recovery may occur sooner than previously suspected. Furthermore, revaccination as early as 3 months following completion of treatment resulted in a protective antibody response for most vaccines as shown by protective IgG levels.

Importantly, all children we studied had completed the pneumococcal vaccination series prior to diagnosis with cancer, six patients had completed the Hib vaccination prior to diagnosis, and 5 out of 7 (71%) had completed vaccinations for diphtheria, tetanus, poliovirus, and MMR prior to their diagnosis; we speculate that previous vaccinations enhanced antibody recover in the post-treatment setting.

Others have shown that damage to the immune system varies as a function of age, type of cancer, and the intensity of chemotherapy [16–18]. However, from our feasibility study, the following factors did not appear to influence the proportion of patients with protective responses against vaccines. Previous studies have shown that younger pediatric patients are at higher risk for developing an inadequate immune response to vaccination [2, 10, 16], but we did not observe this trend in ours. Additionally, we speculate that the shorter duration of treatment and limited use of steroids (as an anti-emetic) may have allowed for better immune recovery in our patient who was treated for Wilms tumor.

Our implementation feasibility study suggests that re-vaccinating non-transplanted children who are off-therapy and in remission for 3 months may be safe and protective. Because resistance to vaccinations continues to challenge our communities, we cannot rely on "herd immunity" to protect off-therapy childhood cancer survivors, calling for further studies in this vulnerable population.
